# Beyond "Two Cultures": Guidance for Establishing Effective Researcher/Health System Partnerships

**DOI:** 10.15171/ijhpm.2016.71

**Published:** 2016-06-13

**Authors:** Sarah Bowen, Ingrid Botting, Ian D. Graham, Lori-Anne Huebner

**Affiliations:** ^1^School of Epidemiology, Public Health and Preventive Medicine, University of Ottawa, Ottawa, ON, Canada.; ^2^Health Services Integration, Winnipeg Regional Health Authority, Winnipeg, MB, Canada.; ^3^Community Health Sciences, University of Manitoba, Winnipeg, MB, Canada.; ^4^eHealth Centre of Excellence, Centre for Family Medicine, Kitchener, ON, Canada.

**Keywords:** Canada, Research Collaboration, Health Research Funding, Partnership Research, Integrated Knowledge Translation

## Abstract

**Background:** The current literature proposing criteria and guidelines for collaborative health system research often fails to differentiate between: (*a*) various types of partnerships, (*b*) collaborations formed for the specific purpose of developing a research proposal and those based on long-standing relationships, (*c*) researcher vs. decision-maker initiatives, and (*d*) the underlying drivers for the collaboration.

**Methods:** Qualitative interviews were conducted with 16 decision-makers and researchers who partnered on a Canadian major peer-reviewed grant proposal in 2013. Objectives of this exploration of participants’ experiences with health system research collaboration were to: (*a*) explore perspectives and experience with research collaboration in general; (*b*) identify characteristics and strategies associated with effective partnerships; and (*c*) provide guidance for development of effective research partnerships. Interviews were audio-recorded and transcribed: transcripts were qualitatively analyzed using a general inductive approach.

**Results:** Findings suggest that the common "two cultures" approach to research/decision-maker collaboration provides an inadequate framework for understanding the complexity of research partnerships. Many commonly-identified challenges to researcher/knowledge user (KU) collaboration are experienced as manageable by experienced research teams. Additional challenges (past experience with research and researchers; issues arising from previous collaboration; and health system dynamics) may be experienced in partnerships based on existing collaborations, and interact with partnership demands of time and communication. Current research practice may discourage KUs from engaging in collaborative research, in spite of strong beliefs in its potential benefits. Practical suggestions for supporting collaborations designed to respond to real-time health system challenges were identified.

**Conclusion:** Participants’ experience with previous research activities, factors related to the established collaboration, and interpersonal, intra- and inter-organizational dynamics may present additional challenges to research partnerships built on existing collaboration. Differences between researchers and KUs may pose no greater challenges than differences among KUs (at various levels, and representing diverse perspectives and organizations) themselves. Effective "relationship brokering" is essential for meaningful collaboration.

## Background

Increasing expectations of greater engagement between decision-makers and policy-makers (potential knowledge users [KUs]), and researchers conducting health services and policy research (HSPR) have led to exploration of factors associated with effective partnerships: many health research funders are developing guidance for health system/academic collaboration.^1-4^

There is a vast theoretical and empirical literature outlining the potential benefits of respectful collaboration, as well as factors and challenges associated with successful partnerships. Several systematic and other reviews have been conducted: while some are generic,^5,6^ others are specific to the health field.^7-14^

However, this research base describes many different kinds of partnerships, and makes different assumptions about who the “partners” are and the purpose of partnership. Some research addresses collaboration among diverse academic disciplines (multi/inter/trans-disciplinary collaboration or “team science”)^15^ while other studies focus on collaborations among health and social care organizations (often for the purposes of enhanced service delivery).

The research focusing on various forms of collaboration between health service researchers and “the community” encompasses both: (*a*) collaboration between academic researchers and specific communities or patient groups (much research is found in the community-based participatory research [CBPR] literature) and, (specific to this study) (*b*) research-related collaboration between academics and various health and social care organizations or systems.

This last category also reflects a broad diversity: some studies address the academic/practice divide, while others focus on collaboration between researchers and policy-makers. Research also reflects different philosophical orientations and objectives for research/KU collaboration.^16^ Partnership objectives are often unclear and range from a commitment to societal equity to the utilitarian goal of promoting greater uptake of research already completed.^17^ Reviews in the HSPR field often assume that the purpose of collaboration is simply to promote use of research findings^18,19^: a goal for which collaboration has been identified as an important strategy.^20^ However, research partnerships for the *purpose* of responding to health system priorities have been less explored.

While many principles and characteristics identified through research with grass-roots community groups are applicable to partnerships between researchers and health organizations (whether at the clinical provider, planning, or policy level), differences in barriers, benefits, and strategies may also be expected. A limited number of studies focus on collaboration in health services and policy^21^: many guidelines for HSPR collaboration are extrapolated from the community collaboration literature.^22^ Different types of partnership encounter different challenges and produce different outputs,^23^ often reflecting the specific context in which the partnership took place. Most studies examine teams that have received research funding: this is only a small proportion of all attempted partnerships.

In spite of this diversity, there is much consensus on potential benefits and commonly experienced challenges. Although there is limited evidence on the impacts of collaboration on patient outcomes or system functioning,^7^ potential benefits of collaboration have been identified as: improved quality of solutions,^6^ greater research relevance and credibility,^18^ enhanced capacity of both researchers and KUs,^10^ greater understanding of partners roles,^18^ personal and professional development,^10^ greater likelihood that research will be applied in practice,^24^ and spin-off benefits such as enhanced skills and networks for future activities.^10,18,25^

Time and resource limitations, challenges of communication, and issues related to power are the most common challenges identified.^6,8,9,11,18,26-28^ Other challenges identified include organizational factors; cultural differences between organizations and disciplines^6^; a need for specialized partnership skills^18^; the inherent complexity of intersectoral collaboration^12^; misalignment of researcher and organizational agendas,^29^ and researcher concern about objectivity and research independence.^18^ Researchers also identify academic reward systems as a challenge to collaboration.^30^

Several conditions associated with successful partnerships have also been identified. Wildridge et al identified 20 critical success factors under the categories of: (*a*) environment (including history of collaboration, legitimacy of leadership, political/social climate), (*b*) membership, (*c*) process and structure, (*d*) purpose (including shared vision and objectives), (*e*) communication, and (*f*) resources.^6^ Sibbald et al identified four characteristics associated with successful partnerships: (*a*) partnership built on existing relationship; (*b*) alignment of researcher/KU agendas; (*c*) participation of skilled researchers, and (*d*) regular multi-modal communication.^26^ Salsberg et al identified the most commonly referenced strategies for effective partnerships as: (*a*) development of an advisory structure; (*b*) development of research agreements, (*c*) use of group facilitation techniques, (*d*) hiring staff from the community of study; and (*e*) frequent communication.^27^ Many authors identify trust and respect, along with opportunities for face-to-face interaction and clear roles and expectations as essential.^9,31-35^ Others stress the need for brokering, boundary-spanning or coordinating roles, as well as skills in change management.^8,36^

While different indicators have been suggested for early and mature partnerships,^22^ research has rarely differentiated between: (*a*) research activities emerging from existing partnerships, and collaborations created for the purpose of the research activity itself; or (*b*) proposals responding to KU priorities, and those soliciting KU support for researcher-initiated proposals. Nor has there been investigation into whether partnerships built on existing partnerships face similar challenges to newly developing ones. Much of the research on partnerships is based on assumptions of researcher-driven initiatives,^37^ often with newly developing partnerships rather than established and supported collaborations. In addition, the voice of KUs is often absent.^34,38^

This paper addresses a gap identified in the literature on collaboration for the purposes of HSPR; and the characteristics associated with success in cases where the partnership: (*a*) is initiated by a health system identified need, (*b*) builds on an existing collaboration, and (*c*) reflects “criteria associated with success” for such partnerships identified in the literature. While Kothari et al suggest partnership indicators to assess the entire life of a collaboration^22^ this paper focuses only on the phase of developing a team, focusing the research question and developing a research grant proposal. This lens is important given the limited proportion of research applications that are successful, and the impact of previous collaborations on subsequent research activity.

## Methods

### Research Objectives

Research objectives were to: (*a*) solicit participant perspectives on, and experience with, on HSPR collaboration in general, (*b*) identify characteristics and strategies associated with effective researcher/KU partnerships, and (*c*) provide guidance for development of effective partnerships.

### Research Context

In 2012, a Steering Committee (SC) (consisting of carefully selected representatives of the provincial department of health, provincial health regions, university education programs, relevant professional organizations, and early adopter sites) was established to lead implementation and evaluation of a primary care innovation in one Canadian province. Funding was obtained to support implementation and implementation evaluation. The project evaluator (SB), experienced in collaborative research and evaluation, together with IB, led a collaborative evaluation designed around KU questions. Design and results of this multi-year evaluation are reported elsewhere.^39^

Based on initial evaluation findings, the SC was successful in obtaining, early in 2013, a Planning Grant (from a federal peer-reviewed research funding program) to explore the feasibility of submitting a proposal to conduct research into several questions emerging from the evaluation. Researchers new to the collaboration joined the partnership at this point: all had previous experience in collaborative KU-led research activities. This grant supported a provincial forum for both KUs and researchers on latest evidence related to the innovation; two separate pre-proposal planning events (summer and fall, 2013) to identify research priorities and discuss strategies for further investigating the innovation; as well as proposal development activities. The proposed research team (consisting of SC members – all but two of whom had been involved in earlier development and evaluation activities – and invited researchers) developed a major funding proposal submitted in late 2013. Research activities described in this paper were conducted with this research team.

### Research Methods

Planning and proposal development activities were designed to reflect guidance for researcher/KU partnerships available in the current literature.

Following proposal submission (early 2014), all signatories to the funding proposal (n = 19) were invited to participate in a confidential semi-structured in-person or telephone interview, conducted by a research assistant (LAH). Questions explored participants’ perspectives on, and experience with, both this specific collaboration, and research collaborations in general (Box 1). Participants were contacted by the Principal Investigator (PI) (IB) by email, who requested consent for LAH to contact them for an interview. Interviews were audio-recorded and transcribed. Interview length ranged from 15 minutes (new team member) to 45 minutes, reflecting both length and intensity of participant involvement in the collaboration.

### Analysis

In order to maintain confidentiality, only SB and LAH had access to the transcripts. Analysis, which used a general inductive approach,^40^ was conducted independently by SB (several readings of transcripts) and LAH (process of rapid identification of themes from review of audio recordings in conjunction with field notes - Rapid Identification of Themes from Audio Recordings [RITA]).^41^ Interview questions provided the focus for cross-case analysis (eg, benefits, challenges, frustrations, suggestions): manual coding using word processing software was employed. Using an open-coding approach,^42^ interview data were then reviewed again for previously unidentified or unexpected themes. Subsequent re-readings of the transcripts focused on overall perspectives of individual participants, noting similarities or differences in responses or perspectives between participants based on role (KU/researcher), experience with previous research initiatives, and relationship with the research topic. Subthemes emerging from initial categories (eg, “frustrations”) were further refined (eg, “time,” “inter-organizational dynamics”) with categories adapted after re-analysis of transcripts. Following independent analyses, SB and LAH met to share results of their analyses, to discuss themes identified, and to ensure that contextual data (eg, intonation, hesitation) only accessible through listening to the audio files was incorporated. No major differences between the two analyses were found, and consensus was reached on themes.

A summary of themes, with many examples of de-identified quotes, was then shared with IB and IG (themselves participants in the interviews, and who were, respectively, leads for KU and researcher team members). Themes were discussed and refined (two iterative meetings) by the three senior authors, focusing on less expected findings and alternate interpretations of data. Learning from the interviews was compared with the current literature in order to develop guidance for future collaborative research activities. Following approval of the draft article by the four authors, the manuscript was circulated to all participants with a request for feedback.

Strategies to help ensure trustworthiness included transcribing interviews verbatim; independent coding by two researchers; inclusion of research and decision-maker perspectives in further refinement of themes; searching for divergent cases; practice of reflexivity (including group discussion of assumptions); and respondent validation (“member-checking”) with research participants.^43^

## Results

Of 19 potential participants, 16 completed an interview. (All who declined were newly invited to the team at the time the proposal was being developed). Four participants were classified as researchers, 11 as KUs, and one as a “hybrid” researcher in a decision-maker role. All researchers and many of the KUs had previous experience in health system research activities, allowing them to compare this activity with other experiences. All KUs were senior decision-makers in the provincial health department, a regional health authority, or the University Faculty of Medicine. Fifty percent were male. While most KUs had been involved since the beginning of the initiative, four joined following initiation of the innovation, and two (1 researcher, 1 KU) were not involved until the stage of developing the major grant proposal. Several team members provided feedback on the circulated draft article: all supported interpretation of the findings.

**Box 1. Interview Guide**

1. Could you confirm for me when you first became involved in the (NAME) initiative? What was your role?
2. Do you feel full use was made of your expertise? (Probe: specifics, suggestions for similar teams).
3. How did you find the experience of developing a research funding proposal in collaboration with researchers/decision-makers?
a. Have you been involved in joint researcher/KU proposal development activities in the past?
i. If yes, in what ways was this experience similar or different?
b. What, from your perspective are some of the benefits of the collaborative approach to research?
c. What were some of the challenges you encountered?
d. Did you ever find the collaboration experience frustrating? (Probes: What would have helped avoid these frustrations? Suggested strategies to avoid/address these challenges?)
e. How did you find communication between decision-makers and managers and the researchers? (Probes: Any difficulties or frustrations?)
f. Were there are any personal or professional benefits to you in being involved in research collaboration? (Probe for specifics).
g. What may have made your involvement more satisfying?
4. What suggestions would you have for similar teams thinking of building a research/KU team for research purposes?
5. Is there anything else you think would be useful for me to know?

After summarizing participant perspectives on the perceived benefits of participatory research, this paper will focus on challenges experienced with established partnerships; and suggest guidance for partnership research built on existing collaborations.

### Benefits of Research Collaboration

Both researchers and KUs expressed overwhelming support for collaborative approaches to research, and expressed similar perspectives on the benefits.

*“Basically that it’s the only way to go. With something that’s trying to do a system redesign with the objective of primary care renewal to not have a collaborative process I feel would really be very short-sighted”* (KU02).

KUs with more research experience were more likely to identify benefits to a collaborative approach, and to comment on differences between various approaches to such partnerships.

*“Some of the projects [I have been involved with] tend to be very, you know, university, sort-of, researcher type focus: “I am doing this.” This was different because it really had the end-user stakeholder piece clearly identified. I think that is the strongest piece of this. You’re actually deliberately having to identify the knowledge users and the people that are involved in the managing of the system. So it creates that link deliberately at the start”* (KU30).

Benefits identified can be classified as: (*a*) research-related impacts, (*b*) organizational/health system benefits, (*c*) benefits to individual participants, and (*d*) benefits to society. Only one participant observed no benefit (*I’m not sure I can see a lot of benefits at this stage.…I think there needs to be a lot more evolution in order to get there)* (KU12).

### Research-Related Benefits

Both KUs and researchers clearly articulated benefits of collaboration to research relevance (that the research is “*meeting a need*”), quality (“*enriches the research methodology and the research itself*” KU11), and likelihood of use (*more likely to be used because the decision-makers have a sense of ownership of it*) (R28).

*“[Without collaboration] in the end, you may not be answering the key questions that they wanted, right?”* (KU10).

*“I think you get better questions and there’s also a quality check because…if you misinterpret the data (Name) is likely to tell you you’ve got it wrong.…And also we’re understanding the limitations of the data before we even start”* (R29).

*“If the researchers had gone off and done this on their own, and we weren’t kind of part of the study in some capacity, we probably would have paid less attention to it and it probably wouldn’t have resonated. We would have wondered, ‘Well did they consider this? Did they consider that? Why did they do this?’”* (KU11).

### Organizational/Health System Benefits

Many placed strong emphasis on the potential organizational/health system benefits of collaboration: Organizational benefits of increased research impact on decision-making and practice, greater likelihood of research use, and enhanced ability to integrate evidence with policy, were commonly discussed.

*“It has much bigger potential for impacting decision-making, and physicians, in the healthcare process.”* (R34).

*“It’s the opportunity to bring research, practice and policy together”* (KU02).

Other benefits identified were earlier opportunities for action; enhanced credibility for organizational decisions; and increased organizational understanding of research and research processes.

*“There’s also opportunity, in a collaboration like this, that you might be able to get some feedback about what’s working and what’s not, during the course of the research project… you’re not necessarily having to wait till the end of two or three years to hear what the research might show. You’ve maybe got a little more insight into what’s working and what’s not”* (KU11).

Perhaps most importantly from the perspective of participants, collaborative research has the potential to provide a supportive environment “*that’s really, really energizing*” (KU15) for creative problem-solving; to provide ‘reflective space’ in an often hectic environment (*“a lot different than what we usually do in healthcare*” [KU11]); and link diverse programs and participants who may not (without the structure provided by the requirements of partnership research) have an opportunity for information-sharing and joint problem-solving.

*“You don’t often get the opportunity to sit down and exchange ideas with people from various [backgrounds], from academic, from leadership, clinicians. We don’t do enough of that in moving the system forward”* (KU07).

*“It really makes you protect time to think about what you’re doing and who your partners are, sort of on the system side…it brings everybody’s eyes to the same problems at the same time”* (R28).

### Benefits to Individual Team Members

Many also highlighted a range of personal and professional benefits to involvement. These included the direct benefits of learning related to research (KU’s) or the healthcare topic (both KUs and researchers):

*“It was very good for me to learn more about, and get some background on [topic of research]”* (R34).

*“I learned a lot along the way about those [research] requirements, coming through it with pretty fresh eyes. To see the approach and the knowledge and the discipline that goes into how to, from an evidence-based perspective, get data. So that’s the thing I’m not often exposed to….I don’t think I had an appreciation for the complexity of the level of rigor and detail that’s required to put a proposal like that together for submission”* (KU15).

Some participants also discussed the benefits of enhanced job satisfaction; exposure to alternate ways of approaching problems; potential career benefits; and development of personal and professional relationships.

*“The fun factor for me has been really good”* (R31).

*“Seeing how the different players or different stakeholders inform that process and how they interact together and work in collaboration in the process”* (KU10).

*“Connections, relationships with people at [the health department] that I wouldn’t have otherwise, that are going to carry over to the future work that I am doing”* (R33).

“*Building connections for new projects*…” (KU30).

“*Benefit…being able to say, or put on your CV, that you’ve participated in a collaborative research project*” (KU11).

### Benefits to Society

Some participants also identified a moral imperative to conduct research that was directly applicable to problems facing both the health system and society, and to be accountable to tax-payers funding the research.

*“Because where either use of our time or our taxpayers’ funds are involved, of which we are stewards, we have an obligation to get the best value out of that”* (KU12).

### Challenges Identified

Barriers to research collaboration have been identified^22,32,34,44,45^ at the individual, organizational, and system level.^46^ This paper, however, reports on collaborative HSPR research that met key partnership criteria (eg, well-developed partnership; involvement of highly placed KUs; infrastructure for collaboration; a successful planning grant supporting both in-person meetings and the organizational administrative time needed; and experienced researchers aware of the logistical demands of partnership grants).

### Time and Resources

Our research confirms earlier findings related to the importance of time (“*the time crunches are really tight*”: [KU30]) as a challenge to meaningful collaboration; however, researchers on the team found that timelines were generally manageable, due largely to the fact that the research had emerged from an existing system-driven initiative with an established structure.

“*I think this team benefited from all sorts of pre-existing relationships and connections and I think that seemed to work very well…There was such a focused issue and focused stakeholders, and there were pre-existing relationships, very good, well-organized, people, good amount of expertise around the table, the decision-makers knew pretty much knew what they wanted….It truly was a manageable thing to do”* (R33).

Nor did resources – at this early stage – present difficulties as the planning grant had supported several collaborative activities, as well as administrative support for proposal development activities.

*“One good thing was we got that planning grant so we could actually have those two meetings and meet with the decision-makers face to face and I think that is really good*” (R34).

Many KUs commented positively on the support provided to meet many of the funding requirements (eg, help create KU CVs). However, “*the complexity of trying to pull together a funding proposal when you’ve got that number of people*,” including scheduling of meetings, was noted as a challenge within the time frames.

### Communication

Consistent with existing research, several issues related to communication were also identified, particularly by KUs. These can be grouped into two major categories: (*a*) orientation to the research process, methods and requirements, and (*b*) communication about progress of the grant. Researchers did not report challenges related to understanding the program and the issues, perhaps because they had the opportunity to review materials on development of the initiative and to attend the provincial forum providing latest evidence on the intervention.

#### Orientation to Research Processes and Concepts

Due to earlier evaluation activities, most participants were well-oriented to the planned proposal, and had participated in both interpreting evaluation findings and determining the research priorities and questions. In spite of this preparation (and dedication during the first planning day to outlining the funding program), many KUs felt that more time was needed on these aspects.

*“I think my advice would be to take more time and kinda walk through [the process]. ‘This is the granting agency we’re dealing with… this is their name…these are the kind of grants that they issue. And these are some of their expectations. And they fund this kind of research but they don’t fund this kind of research. And here’s the deadlines that we’re working towards’…because I think that decision-makers can get kinda lost and get a little confused and maybe don’t want to look stupid so they won’t ask the question”* (KU11).

“*Some terms don’t mean anything - even ones that we didn’t think to define. That label or methodology wouldn’t have meant anything to me”* (KU12).

Researchers agreed that additional time was needed for orientation and capacity building:

“*I also think there’s challenges in general around understanding of what we mean by evaluation, research, performance measurement, accountability, monitoring*” (R28).

#### Communication Throughout the Research Process

In spite of implementation of a multifaceted communication plan (including in-person planning days, follow up meetings, opportunities for input into proposal drafts, updates about submission and anticipated date that results would be announced), several KUs expressed frustration with communication, indicating that more, and more regular, communication is needed than is generally expected by researchers. The gap following communication of successful submission seemed to create the greatest difficulties, even though all were informed of the expected months-long wait time to hear of results.

“*My biggest comment would be, ‘Why don’t I know anything about it?’ You know. How, if you have all these people signing papers and saying thing…I mean I have…submitted my CV for crying out loud, you know? Somewhere in there I’m on this but I know nothing about it*” (KU21).

The challenge of communicating with new members who had not had the benefit of participation in earlier developmental activities was also noted.

*“So the whole issue of you bringing new people on board, and maybe other people drop off, how do you keep that same level of awareness and commitment, is something that I’d look at more”* (R29).

The challenges of both time and communication, however, were experienced as “to be expected” and manageable by most team members.

*“I usually expect that there might be some delays and you might not get the information when you want….There are always difficulties with the language in the way that different people use a bit of different terminology, but those will resolve”* (R34).

Many participants commented on the ‘*respectful*’ environment and lack of observed conflict in these early stages. We found, however, that participant experience in: (*a*) previous research activities, (*b*) factors related to the current collaboration itself, and (*c*) dynamics inherent to the organizational context posed the greatest challenges to the team: these challenges also interacted with time pressures and communication in complex ways.

#### Previous Experience With Research Participation

We found that assessment of the current collaboration was viewed through the “lens” of past research experience.

First, there was often strong concern about traditional “academic” research, and perceived funder support for it.

“*My view of the research community, to be very frank with you, is, quite honestly, extremely self-serving. That researchers believe they have a divine right to explore a topic they’re interested in and that government has an obligation to support them in doing that.…I found it extremely offensive that we think that we should provide 95% of the available funds to sort of pure research, which we can keep uncontaminated by any relevance in application, and then begrudgingly give 5% of it to stuff like [name of funding program]*” (KU12).

Second, previous experience with research supposedly based on “collaborative” principles (but which had not been experienced as such) was observed to contribute to skepticism that affected future involvements (including assumptions that the current project was researcher-driven, despite considerable effort to make it truly collaborative). As one researcher observed:

*“Maybe their past experience with researchers hasn’t been very positive and it’s been, ‘We want this information and you have to do it for us.’ And they thought maybe we were working in that same mode, that it was kind of researcher-driven curiosity stuff as opposed to, ‘Everything we’re asking for it’s because the decision-makers actually need this’”* (R33).

#### Issues Arising From the Established Relationship

We found some unanticipated downsides to building on an existing collaboration. One area of dissatisfaction related to clarity and expectations around roles. The research team proposed the senior provincial and regional decision-makers (co-chairs of the SC for the innovation) as the “key decision-makers” and framed questions around their articulated priorities for health system decision-making. Some, however, did not view this process as sufficiently participatory. Perhaps because of previous involvement in the collaborative evaluation, some KUs had developed expectations of an active participatory role that only became apparent during the confidential interviews. Some stated that they would have appreciated a direct request and discussion about what their role would be, rather than their role being assumed.

*“My personal feeling is that I would have liked to have been more involved, and not just as a knowledge user but recognized as a potential researcher. And I don’t know whether or not that’s appropriate…but it would have made it…would have stroked my ego more….I might have been more involved…not just making comments on the side for adaption. Ya, I think I would have had a higher investment”* (KU03).

*“I think clarity of role and clarity of what the expectations are. ‘So what is your role?’ If you are a decision-maker and get an occasional email, is that it?”* (KU02).

#### Issues Related to Health System Dynamics

In spite of general confidence among partners, dynamics within the health system (among partners at different levels, from diverse sectors, and who had personal and organizational history with each other) created greater difficulties than anticipated. Some issues only became apparent as the result of the interview process, and challenge the common view that primary tensions in research partnerships are those between researchers and KUs.

First, we found some “disconnect” between the priorities of the senior leadership (who had clear ideas of the research questions they wished addressed) and many of the team members (and other staff of partner organizations who were not formally part of the research team but who reported to the senior team members) who were being called on to actually provide information to prepare the grant. This disconnect was experienced by both researchers and KUs, and led to some breakdown of communication.

*“And the other challenge…I mean I understand why this happens, is…you could have a really good discussion with people and they’ll say, ‘Yep,’ that’s exactly what they want, and then they go off and do things, not realizing that what they’re doing is contrary to what they said they wanted.…I believe that they do want those questions asked. But the mechanisms, to operationally make sure that their staff are following their directives seems to be missing. And of course, as the outsiders, we can’t do anything about that”* (R29).

*“I think certain groups [within our organization] have dropped the ball when they were asked to do something”* (KU03).

Second, the complexity of both the healthcare system, and the number of players from different organization also presented some challenges and frustrations.

*“Some of the frustrations on the part of the researchers with complex organizations like healthcare are the many stakeholders and the real difficulty in pin-pointing who has responsibility….There’s sometimes good reason but there’s no one individual who can push a button and make a decision. And I think sometimes [from] the researcher side, it’s just really hard until you’re in the organization, to have an understanding of that. It seems nonsensical”* (KU28).

*“Just trying to get things done…things that would take five minutes…you might be chasing for months….And often…you’re trying to do things collaboratively, but there’s something that has to be done, for example by the region, or by the Minister of Health, or whatever it is, and until that gets done, everything gets held up. And that I find really quite frustrating” (R29).*

Third, we found “behind the scenes” tensions among different levels of the health system, different sectors, and specific individuals. While not necessarily apparent to researchers, these differences in agendas and perspectives appear to create a greater challenge than do commonly assumed differences between the “cultures” of researchers and KUs.

*“You’re framing the questions in terms of sort of a binary, like researchers vs. decision-makers and I think that actually implies a level of homogeneity of those two groups that didn’t exist. There are a bunch of different research perspectives and a bunch of different decision-maker perspectives and just the sheer number of those different perspectives regardless of category made it more complex”* (KU12).

This is not to say that differences between researchers and KU were not recognized.

*“And I think decision-makers, KUs, might often inadvertently disrespect researchers, the rigors of research and the time it takes, because they’re used to working reactive and fast. And then when you start wanting to really dive into proper methods, there isn’t a lot of patience for it. So I think some work still needs to be done on understanding those two worlds and most people don’t”* (KU28).

However, many saw these differences as a source of strength, and understanding of the pressures of the other’s role was often articulated.

*“And it’s not about everyone being homogenized to become researchers…it would be great if they (the decision-makers) learned more about research, but the intent of the project isn’t to make them researchers, or make researchers policy-makers. So co-creating, like helping people understand what the roles are and negotiating those…”* (R31).

Finally, significant frustration was expressed by several KUs about what appeared to be a lack of thoughtful planning by the health system in prioritizing and taking leadership on organizational research priorities, including what was perceived as lack of willingness to commit resources to investigate important health services issues. In the absence of clear processes and partnership criteria, some KUs were inclined to interpret the research initiative as researcher-driven (despite researcher involvement in previous evaluation and planning activities, and the fact that the proposal was designed for, and directly responded to, decision-maker priorities). It even led to some skepticism about the benefits of engaging in applications for research funding.

*“The other question that’s come to my mind is, if it’s not funded does that mean that we should not proceed with some aspects or do we…have a back-up plan if we feel the work’s important enough to inform immediate activity? So I’m just a little concerned that you can then put all of your eggs in the [research funding] basket…And I think that’s a conversation (that) we may want to have”* (KU2).

*“I think researchers trotting off because they’ve made a connection or because somebody’s approached them, I think, you know, checking in early with somebody more senior in the organization to say, ‘We’re interested in studying (X). So-and-so has approached us and has expressed an interest. Is there anybody else we should be working with?’ Because I could see researchers maybe getting engaged or involved with maybe not the most appropriate people, or not necessarily the most enlightened”* (KU11).

These concerns were, on the whole, not evident to researchers who viewed the potential of receiving research funding as wholly positive (“*incentivizing*,” “*they don’t need to pay for [the research]”*).

Our analysis illustrated how the combination of past experiences and dynamics within the health system may present (often unrecognized) challenges to proposal development. For example, senior decision-makers had several clear questions they hoped to address. These priorities were clearly articulated in team meetings, and led to brainstorming by researchers and some KUs about how the research could best be designed in order to address these questions. As criteria were still being finalized for selection of early adopter sites, one suggestion was to select sites based on criteria that would facilitate measurement of factors of concern to KUs. Analysis of interviews revealed diverse interpretations of these discussions: while researchers felt that they were, through brainstorming, attempting to come up with a strategy that would best address KUs needs, this was not the interpretation of all KUs:

*“From the point of view of the researchers, we want a nice distribution of data points so that should enter into the criteria for picking sites. And you know, from my point of view, while I’m totally supportive of research and I think that we want our practice to be evidence informed and one of the ways to do that is to support research, it’s clearly the tail wagging the dog. The objective is to actually advance the health system and an intermediate step to that is to actually get the right providers employed in the right clinics. So, you know, to undermine that objective for the sake of having a nicer mix, a nicer sample from a research point of view was a non sequitur to me”* (KU12).

### Conditions and Activities Associated With Successful Partnerships

Our analysis, confirmed by respondent validation activities, identified several factors perceived as contributing to the creation of successful research partnerships developed in response to decision-maker priorities. These conditions and activities can be categorized under the headings of: (*a*) enabling preconditions for partnership research; (*b*) supporting research team formation; (*c*) promoting team effectiveness; (*d*) facilitating proposal development; (*e*) guidance for proposal submission, and (*f*) guidance following proposal submission.

1.* Enabling preconditions.* Our analysis identified several “preconditions” associated with effective partnerships: pre-existing researcher/KU partnerships; a project selected as a priority by the KUs; appropriate funder requirements and supports; and researcher expertise in collaborative approaches. We also identified particular interest among KUs in greater pro-active planning by health organizations in order to avoid responding to external requests on a case-by-case basis.

2. *Supporting research team formation following an established collaboration.* Even if enabling preconditions are in place, participants identified the need for additional attention, for each specific research activity, to team formation. The following factors were identified:

a. Careful attention to selection of both KUs and researchers for the team. Participants queried how researcher team members were selected: they wanted to work with those who were collegial and responsive to system needs, and wanted to know what expertise they brought to the team. Selection of KU team members was found to be equally important. Our work suggests that the KU team should include the following expertise: decision-making authority related to topic; current involvement with, and knowledge of, the topic; time to attend to the project and to proposal development; credibility with, and access to, key individuals within the organization(s); and – ideally – interest in research.
b. Communication of the rationale for selecting KUs for grant participation to all stakeholders. Participants recognized that it could be challenging to include all key stakeholders in research addressing complex system issues. The importance of ensuring a meaningful role for those involved in day-to-day operations was also stressed.
c. Identification and support for the role of a “relationship broker.” While KUs spoke with frustration about researchers “*trotting off because they’ve made a connection*” (even if this was not the most appropriate one), experienced researchers also recognized the challenge of linking with appropriate KUs, and the importance of a facilitator (a “relationship” broker”) who could assist in determining who they should be partnering with.

*“Having your senior partners, that when there is an issue you can have a chat with them to say… ‘OK, we kind of need help from this part of your organization.’ … So that policy-maker/decision-maker people actually kind of step up when there are barriers or challenges are in the way”* (R31).

Recognition of the time and skills needed to play such a brokering role is also needed.

*“Just recognition that KU engagement is a skill. And you could just say often times researchers are oblivious to, are not as aware of, the organizational culture, and nuances. And so without somebody brokering that relationship, there’s less meaningful input and more barriers to participation”* (KU28).

d. A credible structure to support collaboration. In our case, a province-wide Steering Committee, chaired by senior provincial and regional decision-makers, was the obvious structure to support the collaboration and provided needed credibility. We also identified the importance of jointly developing processes (meeting arrangements, communication strategies) that are realistic and based on KU needs, and ensuring a clear understanding of how administrative support will be provided.
e. Clear expectations, roles and responsibilities of team members. Various team members had diverse perspectives on what was expected of members of the collaboration.

*3. Promoting Team Effectiveness.* We found several factors were associated with team effectiveness:

a. Sufficient time for team development. Many emphasized the importance of opportunities for KUs to provide “real” input into the concept and proposal (to feel that their “*voice was valued*”). This may be even more important to an established team that may have higher expectations of engagement. Very few team members were interested in just “*signing on.*”
b. Development of a respectful environment for positive, collaborative discussion. Many participants commented on the importance to the collaboration of a respectful environment.

*“Creating a climate where everybody’s expertise is acknowledged and respected. So, you know, not having two classes of citizens, like the researchers and then everybody else. [In this activity] whatever their perspective was, it was valued equally.…I’ve been on teams where its sort of been there are ‘Experts’ and there are ‘Other People.’ Even when you’re in the ‘Expert’ category it’s uncomfortable cause you feel badly for the people who may feel badly that they’re, you know, not perceived as the ‘experts’”* (R31).

*“If the Health Economist asks me a clinical question, or I have a Health Economy question that’s, you know, less than a first year basic questions, I don’t know anything about it, that I could feel comfortable asking that question in an environment of such high-power people because that’s not my area of expertise”* (KU7).

c. Common vision and purpose. Participants emphasized the importance of building shared understanding on which the proposal can be built:

*“…a sense of shared vision and shared purpose at the beginning because we have such a diverse participation and diverse location”* (KU15).

*“People to be honest and upfront about what their goals are and I mean, if it’s just to produce a research project, [or] about creating an excellent piece of research that improves policy and healthcare delivery...if we are on that same page to begin with”* (KU07).

d. Opportunities for ‘face to face’ meetings. Many felt that in-person meetings were “*by far the best format for communication because you get way more out it in a shorter period of time*” (KU10). These meetings were felt to bring an “*added level of depth*” and to allow team members to “*really engage in a dialogue*” (KU15).

*“Certainly, one good thing was that we got that planning grant so we could actually have those two meetings and meet with the decision-makers face-to-face.…Quite often you only get to attend meetings by phone or you only get through to some other person and they just sign off…”* (R34).

Others stressed the importance of informal and social events in team building.

*“There were also social events, which I also think are really important to get to know people outside of work….My sense was people were really open and they were listening and asking questions. I think we underestimate the importance of building those personal relationships, because when you’re dealing with researchers and KUs, you need to develop trust. They get to see you - that you’re kind of a normal person and you talk about stuff other than research”* (R31).

While in our case these meetings were possible through specific meeting grant funding, creativity may be needed to find financial support for such in-person activities.

e. Appropriate team orientation. Participants felt that researchers should be prepared to address topics such as: background on the funding program; time lines and deliverables; roles and expectations; support for logistical tasks; key research concepts; and similarities between research, evaluation, performance measurement, and related concepts. KUs should be prepared to provide background on the service/issue, current related initiatives, and to identify any issues that may pose challenges to completing the task.
f. Jointly developed “rules of engagement.” We found that it was necessary to jointly negotiate roles of each team member, communication strategies, costs and compensation for grant development and proposed research activities, data access, ownership of findings and dissemination plans.

*4. Strategies to support proposal development.* Development of a fundable proposal is the first concrete deliverable expected from this preparatory work: we found that the following factors were associated with joint agreement on a research proposal.

a. A research question that is both important to KUs and feasible within the focus of, and funds available through, the grant. A collaborative team must develop mechanisms to work through different perspectives and come to consensus on the research question (an activity that may take much more time than anticipated).
b. Good project management to ensure that funder timelines are met. Participants commented on the importance of adequate logistical support in ability to ensure deadlines were met.
c. An effective relationship broker. Although time constraints risk making this phase simply task oriented, participants highlighted the importance of a relationship broker role to flag potentially sensitive areas or misunderstandings, negotiate relationships between team members with diverse perspectives, and facilitate communication.

*“So yeah, there was a lot of, some conflict I guess, quiet conflict. But I think there’s some brokering at the coordinator role, a ton of work brokering that. The role itself is a kind of make-or-break role and if you’re not good at it, you could really break it. Like how to get hold of the CEO [Chief Executive Officer] of an organization and bring them on board quickly, and get their CV in there and their buy in…communicating with the right people in the right way. Logistics. Understanding the culture in which they work, respectful engagement”* (KU28).

This role is also necessary to ensure that the there is support for the (often) complex logistical requirements of grant development and submission: ensuring that KU have support to complete such tasks as CV development.

*“I think without that support, I would have found it very challenging. So I think for people like me, that aren’t as exposed to the rigor of those requirements, that would be essential. That there be that capacity-building for it and helping people understand what the next steps in the process were”* (KU15).

*5. Activities following proposal submission.* Finally, our experience suggests that attention is required to support ongoing collaboration after the proposal is submitted and team members have been notified. The 6-8 month waiting period for the funding decision often results in KUs “forgetting” details of the grant, given the many other tasks and issues they are addressing at any point in time.

*“There were long periods where you didn’t hear much about, you know, where’s this thing at? And it could be because there was nothing going on”* (KU30).

## Discussion

There are a number of limitations to this research. First, it interviewed team members from only one initiative (with the specific partnership characteristics noted earlier), in the Canadian healthcare setting. In addition, the research activities described were being developed in the context of multi-level system wide reform: although activities were focused on one innovation, many other inter-related primary care initiatives were underway at the same time. While findings may not, therefore, be directly transferrable to smaller, project-based activities, they provide focused insight into the challenges of collaborations that meet the criteria for effective partnerships identified in the literature, and where partnerships are developed around decision-maker priorities: a current knowledge gap. They also address another common gap in the research: the failure to include the voices of KU partners.

Our research confirms many of the findings identified in the literature on potential research benefits of collaboration. We also found a number of reported benefits related to what is often referred to as “capacity building.”^10^ Benefits in this category appear not to be limited to directly research-related issues. Rather, both researchers and KUs emphasized the learning that resulted from exposure to diverse skills and perspectives (ways of looking at problems): this transferable learning may be one of the greatest benefits to collaboration.

Perspectives on time and resource limitations and communication barriers were similar between researchers and KUs in this setting. However, perhaps because the researchers interviewed were experienced in collaborative research, most challenges highlighted in the literature (related to planning the research, timelines, data issues, and communication among KUs) while experienced, were largely expected: they were seen as part of the job and did not pose serious obstacles (a similar finding to that of Sibbald et al).^26^

Our research has highlighted, however, additional potential challenges related to past experience with research, history of the existing collaboration, and dynamics among KUs themselves, illustrating how these dynamics may play out in development of research proposals. While building on an existing collaboration may provide the research partnership with a strong foundation, past history may contribute to additional challenges. In such situations, it is important to recognize the expertise of individual KUs and explore with them potential roles and anticipated contributions; to be clear about roles and expectations; and to develop strategies to orient any members recently added to the partnership.

While organizational culture has been identified as a factor in promoting effective partnerships,^6^ this research identified the importance of both organizational complexity, and the diversity of inter and intra-organizational perspectives. While not always visible to researchers, these dynamics may have an enormous impact on collaborative research activities.

Our findings suggest that the primary tensions in partnership research may not always be between researchers and decision-makers (the so-called two-cultures hypothesis; positing that researchers and decision-makers live in two very different worlds, with different objectives, expectations, and ways of operating).^47^ This hypothesis has led to the creation of the role of ‘knowledge broker’ (KB), intended to interpret between the two cultures. Our work with an established KU/researcher team, however, questions the usefulness of this division, as differences in cultures (and agendas) were found as much among KUs as between researchers and KUs.

This research also highlights the importance of a knowledgeable “relationship” broker (as distinct from a knowledge broker), who can navigate, not simply between researchers and KUs, but among potentially competing KU agendas and relationships, both within the team, and across different organizations. Many authors have identified the importance of some form of “boundary spanning” role in effective collaborations,^48,49^ and illustrated the diverse roles that so-called knowledge brokers (KBs) may play. To date, however, much emphasis on such roles has been on transferring knowledge between various stakeholders rather than facilitating relationships, and the diversity of roles has not allowed for confident assessment of benefits.^48^ Reframing the role to focus on “relationships” rather than “knowledge” may be useful. To be effective, the relationship broker requires in-depth knowledge of organizational culture(s); knowledge of the individual ‘players’ associated with the issues; coordination resources to help ensure timelines are met; protected time; and research experience.

Coordination of so-called collaborative research proposals is, however, often managed from within an academic centre by a research assistant (often a student who may or may not have this experience) rather than by hiring staff from the community of study. Our experience supports previous research suggesting that situating such a role within the partner organization (possible in our case through planning grant funds) is more likely to be effective; and may be a consideration in setting criteria for collaborative funding programs.

This research also raises implications for research funders. Inclusion of KUs may present additional time, logistical and interpersonal challenges. Research proposals focusing on *system change* require broad engagement, suggesting that it may be useful for funders to reflect this complexity in RFP guidelines, timeframes, evaluation criteria, and targeted funding programs to support collaborative grant proposal planning. Without the planning grant support for relationship and proposal development activities (from which this activity benefited), true engagement would not have been possible – even given an established partnership – in the available time frame.

Wait times inherent in grant timelines of many major funders pose challenges to collaboration: creative strategies to facilitate timely decisions on decision-maker initiated grants are needed. Also of potential interest to research funders is the strong emphasis given by participants to organizational and health system benefits of collaborative research. This may serve a reminder that, in measuring research impact, it is important to look beyond direct utilization of research findings, and attempt to capture benefits arising from the research process itself.^50^ While many frameworks outlining categories of research impact have been developed,^51-55^ recent research suggests that in practice these indirect benefits may be undervalued.^56^

After several years of “collaborative” research funding opportunities, many KUs have experience with how such collaboration is generally interpreted and managed by researchers. Requirements by major funders for researchers to have KU partners have created an environment where many researchers feel they must form partnerships with knowledge-users even if this is not their interest. Consequently, many KUs have had experience with so-called collaborative research that has coloured their expectations of future partnerships. Few may have experience with authentic research collaborations driven by decision-maker priorities rather than researcher interests, creating challenges for researchers interested in developing a research agenda around KU priorities. Researchers committed to collaborative research are advised to develop strategies to assess potential partners’ past experience, and explicitly outline their assumptions and motivations for collaboration. While KUs in our study remained supportive of the principle of collaborative research, they also expressed a number of frustrations about the current funding environment, as well as common research practice. This suggests that the current review process may not be optimally effective in recognizing (or giving appropriate weight to) genuine collaboration in response to health system needs.

Integration of our research with the existing literature on research collaboration suggests a number of recommendations for developing successful research partnerships in response to KU priorities. This guidance is relevant for KU organizations, researchers, and research funding bodies. Tables 1-4 revisit factors identified in the section *Conditions and Activities Associated with Successful Partnerships* in order to provide concrete guidance for specific early stages of partnership development. Table 1 identifes enabling conditions for partnership research; Table 2 provides guidance for team formation; Table 3 provides guidance for promoting team effectiveness; and finally, Table 4 presents guidance for proposal development by collaborative teams.

**Table 1:** Enabling Preconditions for Partnership Research

**Preconditions**	**Rationale**	**Implications for Funders**	**Implications for Health System**	**Implications for Researchers**
Pre-existing researcher/KU relationships	As trust requires time to develop, established relationships facilitate team development. Many issues will have been addressed, allowing more time to focus on research plan.	- Fund opportunities for joint learning and planning - Ensure review processes assess and recognize established partnerships	‏- Develop guidelines for partnership, clarifying requirements and expectations of academic partners - Initiate contact with academics interested in partnership	‏- Begin establishing relationships with KU in areas of interest to you before initiating research - Learn about relevant organizations/programs
Project identified as priority by KUs	Research that responds to KU concerns is more likely to address priority health needs, have results used, and facilitate partnership development process.	- Ensure that proposal evaluation processes identify and recognize proposals responding to KU driven research	- Develop organizational and program priorities for research and research partnership	Clarify KU priority issues, and explore your potential contribution
Appropriate funder requirements, supports	Funding programs vary in lead time; weight given to KU partnerships; and pre-proposal support for planning.	‏- Review funding requirements to facilitate true partnership	- Explore “Planning” grant opportunities to fund initial developmental work - Ensure organizational contribution responds to identified priorities and is appropriately resourced	‏- Explore funder criteria and requirements carefully to ensure funding program supportive of partnership work - Explore “Planning” grants to fund initial developmental work
Researcher expertise in collaborative research approaches	Many researchers have little education or experience in partnership approaches. Research experience and commitment is predictive of partnership success.	- Support strategies to develop researcher skills in partnership research - Recruit experienced collaborative researchers to planning and review committees	- Be explicit about partnership expectations - Proactively identify researchers with partnership experience & approaches - Interview prospective partners about what partnership means to them	- Explore opportunities to gain experience within the health system - Explore collaborations with experienced partnership researchers - Develop skills, such as evaluation research, in researching ‘real-life’ questions

Abbreviation: KU, knowledge user.

**Table 2:** Guidance for Team Formation

**Criteria Associated With Success**	**Rationale**	**Implications for Funders**	**Implications for Health System**	**Implications for Researchers**
Careful selection of KUs and researchers for the team	Team members must have the appropriate knowledge and skills to contribute to the research question and to work in partnership. In addition, KUs must be in a position to facilitate access and action.	- Ensure proposal review process includes criteria to assess whether all relevant KU and researchers are included on the team, and that skills and roles of all team members are appropriate - Require researcher applicants to identify KU roles on grants and publications on their CVs	- Select KUs with decision-making authority related to the topic; current involvement with and knowledge of the topic; time to contribute to the project; credibility with and access to key individuals within the organization; and willingness to work with researchers	- Ensure researcher team members have: (*a*) relevant methodological expertise, and (*b*) interpersonal skills to work with KUs - Develop understanding of roles and potential contribution of key players in partner organization(s)
Clear communication of rationale for selecting KUs for grant participation	Clear communication about decision-making processes is essential in ensuring organizational confidence in research activity.	- Support development of guidelines for team composition	- Share organizational partnership In collaboration with research lead - Hold individual discussions with selected KUs on their role, interests, and time implications - Provide additional orientation to those new to the collaboration - Ensure all stakeholder groups are aware of planned research and its importance to decision-makers	- In collaboration with KU lead, undertake individual discussions with potential KUs re: roles, interest and responsibilities - Proactively identify orientation needs of current and new team members
Identification of and support for “relationship broker”	The health system (and individual health organizations) is complex, with diverse expertise, roles, agendas, authority, and mandates. Understanding this diversity, combined with skill in negotiating diverse perspectives and agendas, is needed.	- Ensure the proposal review process considers the engagement and alignment of relevant organizational groups needed for the project to be successful, including identification of individuals to broker relationships - Recognize, in proposal review process, benefits of such roles placed within KU organizations	- Identify an appropriate person to serve as project relationship broker - Ensure this person has authority to engage and/or trouble shoot across organizational units, skills to manage boundary-spanning roles, and protected time to provide leadership	- Encourage and support selection of this role - Develop an effective relationship with the relationship broker - Engage the relationship broker to explain the workings of the organization and the organizational perspective
Credible structure to support the collaboration	Good project governance is critical both to meet timelines and ensure appropriate participation and communication.	- Ensure review process assesses appropriateness of project governance and other strategies to meaningfully engage KUs with decision-making authority in the topic area - Ensure funding guidelines for eligible expenses support appropriate collaboration expenses	- Ensure appropriate decision-makers across organization are engaged (at relevant level in the organization) - Understand implications of the project on operations and manage accordingly	- Work with KUs to establish mutually acceptable processes (meetings, communication, etc) - Provide support (HR, cash, in kind) to selected structure and processes
Clear expectations, roles and responsibilities	A clear understanding of roles and responsibilities can increase confidence of team members and facilitate effective team functioning.	- Ensure review process considers project team’s strategies for ensuring clarity, their feasibility and likelihood of success	- Ensure early discussion of expectations, roles, responsibilities, and resources required to manage the project - In collaboration with research lead, ensure documentation of expectations, roles and responsibilities - Clarify processes for identifying and resolving any issues, including strategies for conflict management	- Work with KUs to reach consensus about expectations, roles and responsibilities, and strategies for dealing with disagreements/tensions - Clarify processes for identifying and resolving any issues, including strategies for conflict management - Document in writing all decisions re: expectations, roles and responsibilities - Revisit expectations, roles and responsibilities during the project

Abbreviations: KU, knowledge user; HR, human resource.

**Table 3:** Guidance for Promoting Team Effectiveness

**Criteria Associated With Success**	**Rationale**	**Implications for Funders**	**Implications for Health System**	**Implications for Researchers**
Sufficient time for team development	Time is needed to develop relationships and trust among partners.	- Provide funding opportunities for planning and development activities	‏- Ensure that KUs on team have protected time to participate in relationship building activities	- Dedicate time for relationship development
Respectful environment for positive, collaborative discussion	Even when KUs and re-searchers know each other, conscious efforts are needed to create a safe environment that facilitates respectful discussion and exchange. This is even more important when the parties are new to each other.	‏- Ensure review process considers tone of proposal (Does it reflect respect for KUs as equal partners? What is tone in letters of support?)	- Create needed space and time for KUs to participate in discussions with researchers - Establish expectations about collaboration and respect for diverse perspectives, expertise and skills - In collaboration with researchers, clearly identify areas of expertise of each team member	- Acknowledge expertise and skills of KUs and demonstrate that they are valued and important for the project - Demonstrate your interest in learning from KUs - Ask KUs about questions they may have: reassure that there are no “dumb” questions - In collaboration with KUs, clearly identify areas of expertise of each team member
Common vision and purpose	Without a common vision, a project is unlikely to be successful.	- Ensure review process evaluates extent to which KU support letters suggest common vision, purpose and understanding of the project	- Collaboratively with researchers, develop strategies to come to consensus on purpose (research questions) and vision for the project - Ensure sufficient time for such discussions - Articulate desired goals and outcomes from the organizational perspective	- Collaboratively with KUs, develop strategy to come to consensus on purpose (research questions) and vision for the project - Allocate additional time in planning processes for these discussions
Opportunities for “face-to-face” meetings	Research indicates that in-person contact is more effective in building effective working relationships.	- Provide funding for planning meetings - Ensure review process considers whether and to what extent the team has met and truly collaborated on the proposal development	- Provide leadership in identifying appropriate meeting times for KU partners - Encourage and facilitate attendance of all KUs - Support opportunities for social contact (eg, dinner meetings)	- Plan meetings at time and place convenient for KUs - Consider use of development funds to support opportunities for social interaction
Appropriate team orientation	In order for diverse participants to form a team, it is necessary that they under-stand the perspectives, expertise, and contribution of each party; and that the team has the opportunity to develop shared understanding of key concepts.	‏- Ensure review process considers work plan to ensure that orientation needs are considered	- Proactively identify areas where KU orientation is needed - Provide researchers with background they need to understand research context - Provide background on service/issue, related initiatives, and identify any issues that may pose challenges to completing the task	- Describe funding opportunity, timelines, deliverables, roles and expectations of grant proposal (eg, completion of a CV), support for logistical tasks, key concepts, similarities and differences between performance measurement, research, evaluation, etc. - Identify and prepare to address KU questions on methodology
Jointly develop “rules of engagement”	Teams are likely to be more effective, and avoid misunderstandings if potentially sensitive issues are negotiated in advance.	‏- Support development and dissemination of resources aimed at increasing awareness and skills in how to conduct collaborative research	‏- In collaboration with researchers, determine roles of team members, communication strategies, costs and compensation for grant development and proposed research activities, data access, ownership of findings, dissemination plans, and strategies for conflict management - Ensure organizational concerns addressed	- In collaboration with KUs, address joint issues - If existing organizational group becomes the coordinating structure, adapt to existing team culture and find appropriate ways to participate

Abbreviation: KU, knowledge user.

**Table 4:** Guidance for Proposal Development

**Criteria Associated with Success**	**Rationale**	**Implications for Funders**	**Implications for Health System**	**Implications for Researchers**
Identification of important, researchable, feasible question	The purpose of collaborative research is to address research questions of importance to KUs. The proposal must also meet grant criteria and be feasible within the funds available.	- Support planning grants to promote collaborative development of useful questions - Include in application process request for evidence that research question is key priority for KUs (eg, letter of support from KUs) - Include in review process reviewer guidelines to assess relevance and potential impact of the project, its feasibility, and adequacy of funding requested to complete the task	- Establish KU organizational process for prioritizing research questions of relevance to health system - Develop, in collaboration with affected partners, mechanisms to reach consensus on the research question - Clarify areas of expertise (research, program) for formulating question - If questions have been determined by one or more senior decision-makers, ensure that rationale for decision is clearly communicated within the organization	- In collaboration with KU partners develop mechanisms to reach consensus on the research question - Clarify areas of expertise (research, program) for formulating question
Good project management practice	The complexity, and short time lines of many grant opportunities require efficient time management and good coordination.	- Support planning grants to enable prospective teams to develop needed infrastructure	- As a team, develop work plan with time lines and assigned responsibilities - Identify and negotiate needed organizational logistical support before beginning task	‏- As a team, develop work plan with time lines and assigned responsibilities - Consider use of development or other funds to support work best conducted from within the partner organization(s)
Support for role of identified relationship broker	Time constraints risk making this phase simply task oriented. A relationship broker is needed to flag potentially sensitive areas or misunderstandings, negotiate between diverse perspectives and competing priorities, and address roadblocks in a timely way.	- Ensure funding guidelines recognize importance of funding such individuals in KU organizations and recognize related costs as eligible expenses	- Ask for regular updates from the relationship broker to identify potential areas of difficulty, and specific areas where KUs may need assistance (eg, developing CVs) - Provide support in addressing any identified problems - Ensure relationship broker has direct access to decision- makers	- Work with relationship broker to understand KU organization(s) and cultures - Ask for regular updates on tasks to be completed by organizational members, and any difficulties encountered

Abbreviation: KU, knowledge user.

## Conclusion

This paper is one of the first to explore both KU and researcher experience with participation in collaborative HSPR initiatives: (*a*) driven by KU priorities, and (*b*) based on an existing partnership. A number of practical guidelines for developing research proposals in such an environment are proposed: these build on existing literature and reflect the findings that while (in established collaborations) many challenges are manageable, previous experience with research activities, factors related to the established collaboration, and interpersonal, intra-, and inter-organizational dynamics may pose unanticipated challenges. The previous focus on addressing challenges related to differences in the researcher-KU role should be expanded to strategies for responding to differences in roles, perspective and agendas of all team members.

## Acknowledgements

This work is supported by a Canadian Institutes of Health Research (CIHR), Ottawa ON, Canada Planning Grant [RFN #126820]. In addition, Ian D. Graham is the recipient of an inaugural CIHR Foundation Grant [FDN #143237].

## Ethical issues

This study was approved by the University of Manitoba Health Research Ethics Board, Winnipeg, MB, Canada file number HS15442 (H2012:222).

## Competing interests

Authors declare that they have no competing interests.

## Authors’ contributions

SB designed the study in collaboration with IB and IG. Interviews were conducted by LAH. SB led the analysis and interpretation, in collaboration with all authors. SB drafted the manuscript IB, IG, and LAH provided critical review and revisions. All authors approved the manuscript.

## Authors’ affiliations

^1^School of Epidemiology, Public Health and Preventive Medicine, University of Ottawa, Ottawa, ON, Canada. ^2^Health Services Integration, Winnipeg Regional Health Authority, Winnipeg, MB, Canada. ^3^Community Health Sciences, University of Manitoba, Winnipeg, MB, Canada. ^4^eHealth Centre of Excellence, Centre for Family Medicine, Kitchener, ON, Canada.

## Key messages

**Implications for policy makers**

- *“Relationship brokering”* may be a more useful concept than *“knowledge brokering”* in promoting and supporting established researcher/knowledge user (KU) partnerships.
- Differences between research and KU cultures identified in the literature may pose no greater challenges to collaboration than differences among KUs (at various levels, and representing diverse perspectives and organizations) themselves.
- Experienced teams often find commonly identified challenges to collaboration (eg, communication, time) manageable.
- Health organizations that proactively determine their research priorities and criteria for research engagement are likely to experience more satisfactory research partnerships.
- Effective health services and policy research (HSPR) partnerships are encouraged by funder support for planning and preparatory activities; recognition of health system costs and contributions; responsiveness to KU time constraints; and strategies to identify and support system-driven health initiatives.

**Implications for public**

The health system is facing complex challenges that require collaboration between researchers, policy-makers, and practitioners. One of biggest challenges is the development of collaborative researcher/knowledge user (KU) teams to develop and conduct relevant research. This research identifies guidelines for promoting effective research partnerships. More effective partnerships will increase the likelihood that research conducted to improve healthcare delivery will address important questions and be useful in practice.
